# Glucosamine for Osteoarthritis: Biological Effects, Clinical Efficacy, and Safety on Glucose Metabolism

**DOI:** 10.1155/2014/432463

**Published:** 2014-02-11

**Authors:** Juan Salazar, Luis Bello, Mervin Chávez, Roberto Añez, Joselyn Rojas, Valmore Bermúdez

**Affiliations:** ^1^Endocrine and Metabolic Diseases Research Center, Faculty of Medicine, University of Zulia, Maracaibo 4004, Venezuela; ^2^Institute of Clinical Immunology, University of Los Andes, Mérida 5101, Venezuela

## Abstract

Osteoarthritis is a chronic degenerative disorder that currently represents one of the main causes of disability within the elderly population and an important presenting complaint overall. The pathophysiologic basis of osteoarthritis entails a complex group of interactions among biochemical and mechanical factors that have been better characterized in light of a recent spike in research on the subject. This has led to an ongoing search for ideal therapeutic management schemes for these patients, where glucosamine is one of the most frequently used alternatives worldwide due to their chondroprotective properties and their long-term effects. Its use in the treatment of osteoarthritis is well established; yet despite being considered effective by many research groups, controversy surrounds their true effectiveness. This situation stems from several methodological aspects which hinder appropriate data analysis and comparison in this context, particularly regarding objectives and target variables. Similar difficulties surround the assessment of the potential ability of glucosamine formulations to alter glucose metabolism. Nevertheless, evidence supporting diabetogenesis by glucosamine remains scarce in humans, and to date, this association should be considered only a theoretical possibility.

## 1. Introduction

Osteoarticular disease currently represents one of the most common presenting complaints in clinical practice, generating severe impacts in the quality of life of patients and representing a heavy economic burden for public health systems [1]. Within this group, osteoarthritis (OA) is the most prevalent articular disorder, with a prevalence of up to 80% in subjects over 65 years of age [2]. Nevertheless, variability in diagnostic criteria, variations of methodology in epidemiologic studies, and a relative scarcity of research in the subject have obscured the true scope of this issue [3].

Venezuela does not escape this scenario. As described in epidemiologic reports by the National Center of Rheumatic Disease during the 1995–2010 period, OA is the third osteoarticular disorder most frequently diagnosed at first consultation, representing 16,22% (*n* = 13,983) [4]. Still, said report does not specify whether these figures include diagnoses made in primary attention consultations, which should ideally detect most of these patients.

OA, also called osteoarthrosis, is a chronic arthropathy characterized by the degeneration and loss of articular cartilage, resulting in disruption of its mechanical properties and those of subchondral bone as well as modifications in the surrounding soft tissue. Although this process can develop in all osteoarticular structures, the knee remains the most accessibly assessed and the one with the most specific evaluation criteria [3, 5]. Nonmodifiable risk factors associated with OA include advanced age, female gender, and ethnicity; while articular overload, intense physical activity, and obesity are modifiable risk factors. Altogether, these components must all be included in the integral management of this kind of patients [5].

Currently recognized pathophysiologic mechanisms involve not only articular lesion and degeneration but also a coexisting chronic inflammatory process which favors the progressive loss of hyaline cartilage through numerous molecular mediators [6]. Furthermore, chondral structures may not be the sole target of this deterioration, since all components of the articular surface seem to be affected [7]. In recent times, this paradigm shift has led to an ongoing review of therapeutic management schemes for these patients, wherein glucosamine supplements remain cornerstone prescriptions in clinical practice, both by primary attention of physicians and specialists [8]. Nevertheless, their biochemical features and implications render it necessary to delve further into their repercussions over carbohydrate metabolism, considering the array of endocrine-metabolic adverse effects they have been linked to.

## 2. Pathophysiology of Osteoarthritis

Despite the great prevalence and impact of OA in the adult population, its specific etiology remains unknown; and much like most chronic diseases, a constellation of risk factors have been proposed to interact amongst each other in this case, both biochemically and mechanically, ultimately leading to the onset and progression of this disease [9]. Articular cartilage, a fundamental component of the osteoarticular system, is the main degradation target, yet other structures are also affected including subchondral, capsular, synovial, and periarticular soft tissue [10, 11]. Still, the principal disruption occurs within the chondrocyte, with an unbalance between the synthesis and degradation of extracellular matrix, because of an excessive local release of proteolytic enzymes, and a progressive deceleration of cartilage reparation [12, 13].

In addition, a vast catalogue of bioactive molecules is synthesized at the chondral level, including proinflammatory cytokines IL-1, IL-8, IL-17, IL-18, and TNF-*α*, as well as free radicals (nitric oxide), growth factors (TGF-*β*), and lipidic mediators (Prostaglandin E_2_, Leukotriene B_4_) [14]. This inflammatory component develops primarily at the synovial membrane, coexisting with other degenerative mechanisms, and has led research efforts to contemplate therapeutic interventions directed to the stimulation of cartilage synthesis, modulation of inflammation, and regulation of chondrocyte metabolism [6, 15].

**Figure 1:**
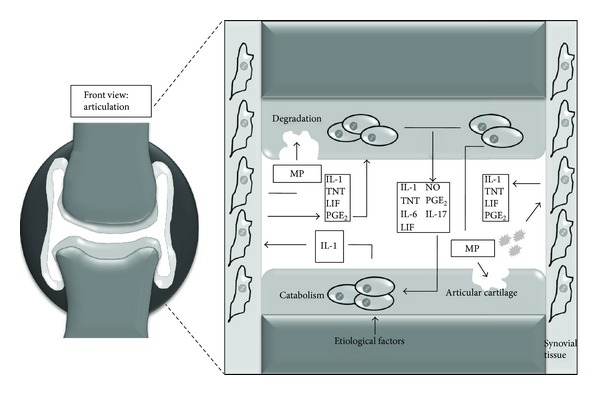
Physiopathology of osteoarthritis. IL: interleukin; TNF: tumoral necrosis factor; NO: nitric oxide; PG: prostaglandins; MP: metalloproteases; LIF: leukemia inhibitory factor. Targets of diverse pathophysiologic factors of osteoarthritis include not only articular cartilage but also several structures of the articular surface, where an unbalance favoring catabolism occurs, with degradation of extracellular matrix. This process is triggered by numerous proinflammatory and proteolytic molecules which generate a local vicious circle. (Refer to text.)

Notably, Aspden et al. [16] have suggested considering OA as a systemic disease, where the main disruption would involve lipid metabolism and stromal cell differentiation, a concept stemmed from the common embryologic origin shared by all structures constituting the articular cavity. Nonetheless, current views remain focused on the local pathology, where novel pathophysiologic pathways and factors are constantly discovered, generating potential therapeutic targets [17]; Figure 1 depicts the main pathophysiologic routes of OA.

## 3. Therapeutic Management of Osteoarthritis

Along with pharmacologic agents, nonpharmacologic measures remain a cornerstone of OA treatment, fundamentally, the management of all risk factors involved and possible comorbidities such as obesity, diabetes, and menopause [18]. Therefore, patient education, physical activity, physiotherapy, articular protection, postural hygiene, and weight control are essential injury- and pain-limiting tools, which are generally included in all clinical guidelines for the integral management of OA patients, albeit receiving varying degrees of recommendation throughout different regions worldwide [5, 18–21].

On the other hand, the main objective of drug use in OA is symptomatic management, reducing both pain and underlying inflammation [22]. Various management guidelines have categorized these drugs as Symptom Modifying Osteoarthritis Drugs (SMOADS) [23, 24] which are divided into 2 subgroups:

- rapid-acting drugs including analgesics, nonsteroidal anti-inflammatory drugs (NSAIDs), and intraarticular glucocorticoids and opioids;
- slow-acting drugs or SYSADOA (Symptomatic Slow Acting Drugs for Osteoarthritis).

Regarding the first subgroup, paracetamol is considered the initial drug for the management of knee OA [25, 26], with NSAIDs being broadly recommended if no satisfactory results are observed after first-line management, although their adverse effect profiles should be considered prior to prescription [27, 28]. Lastly, intra-articular opioids and glucocorticoids are only implemented in very specific situations where initial treatment has been inefficient [19]. In general, utilization of drugs in this subgroup depends on their safety profile, patient consent, cost-effectiveness, and other factors relevant to the specific clinical evolution of patients [5, 23].

**Figure 2:**
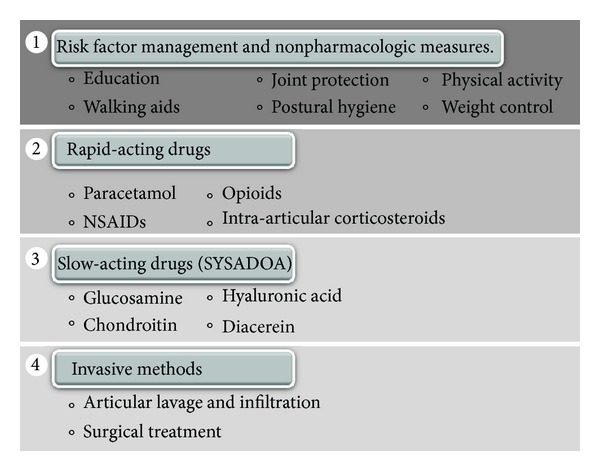
Possible interventions in therapeutic management of Osteoarthritis.

Findings reported in the 90s decade about articular cartilage, its metabolic activity, and regenerative capacity [29, 30] have led to the proposal of chondromodulating and/or chondroprotective substances, which constitute the group of slow-acting drugs or SYSADOA, including cartilaginous matrix precursors (glucosamine, chondroitin, and hyaluronic acid) and cytokine modulators (diacerein and metalloprotease inhibitors) [24]. These drugs, particularly glucosamine (GluN), have raised controversy regarding their utilization, due to inconsistencies in findings on their effectiveness in the treatment of these patients [19, 31, 32].  Figure 2 summarizes the therapeutic management of OA patients.

## 4. What Is Glucosamine? Molecular Aspects

**Figure 3:**
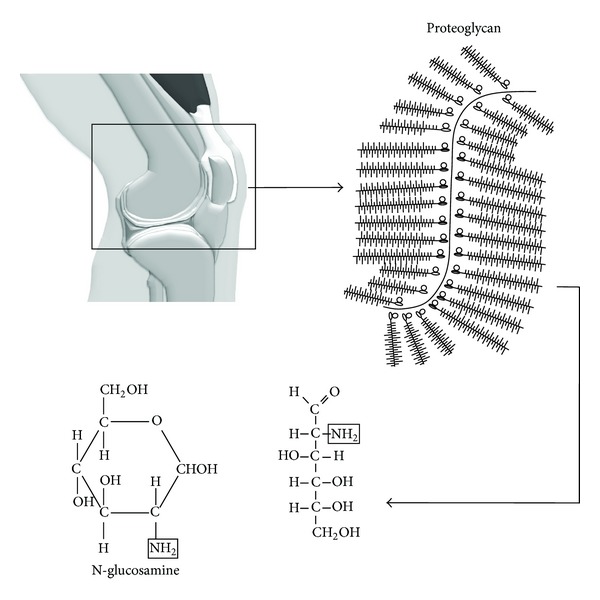
Chemical structure of glucosamine.

GluN (2-amino-2-deoxy-D-glucose) is an amino-monosaccharide derived principally from chitin, a compound found in the exoskeleton of certain marine invertebrates [33]. GluN is an essential noncellular component of connective tissue, cartilage, ligaments, and other structures [24, 34] (Figure 3). The main compounds including GluN are glucosamine hydrochloride, glucosamine sulfate, and N-acetylglucosamine [34]. The latter can be organically synthesized through the hexosamine pathway, an alternative metabolic route to glycolysis, which is esteemed to consume up to 5% of glucose in adipocyte cultures [35].

This metabolic pathway is essential for the biosynthesis of amino sugars, utilizing fructose-6-phosphate and glutamine (as an amino-group donor) to produce glucosamine-6-phosphate (GluN-6-P), catalyzed by the enzyme glutamine:fructose 6-phosphate amidotransferase (GFAT), which represents the rate-limiting step in this process [36, 37]. Besides this “endogenous” production, glucosamine provided exogenously can be introduced to cells through glucose transporters (especially GLUT-2), and phosphorylated intracellularly by hexokinase to GluN-6-P, avoiding the rate-limiting reaction of the aforementioned pathway [38, 39]. The next step is the acetylation of GluN-6-P to N-acetyl-glucosamine 6-phosphate (N-Acetyil-GluN-6-P), catalyzed by glucosamine-phosphate-N-acetyltransferase. Then, this compound is transformed into uridine-5-diphosphate-N-acetyl-glucosamine (UDP-N-Acetyl-GluN) by the enzyme UDP-N-acetyl-glucosamine pyrophosphorylase. UDP-N-Acetyl-GluN is the precursor for the biosynthesis of amino sugars which serve as building blocks for GAGs, proteoglycans, and glycoproteins, by transferring **β**-N-acetyl-glucosamine to the hydroxyl groups of serine and/or threonine residues of a broad span of proteins [34, 40, 41]. Lastly, UDP-N-Acetyl-GluN can be converted to UDP-N-acetyl-galactosamine through isomerization mediated by the enzyme N-acetyl-glucosamine-4-epimerase [37].

**Figure 4:**
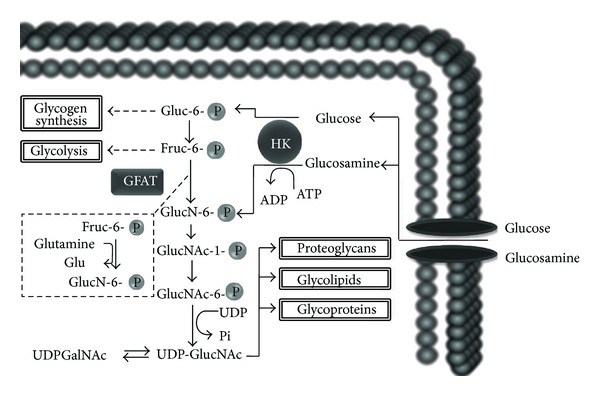
Glucosamine biosynthesis. HK: hexokinase; Gluc-6-P: glucose-6-phosphate; Fruc-6-P: fructose-6-phosphate; GFAT: glucosamine fructose-6-phosphate amindotransferase; GlucN-6-P: Glucosamine-6-phosphate; GlucNAc-6-P: N-acetyl-glucosamine-6-phosphate; GlucNAc-1-P: N-acetyl-glucosamine-1-phosphate; UDPGalNAc: uridine diphosphate (UDP)-N-acetyl-galactosamine; UDP-GlucNAc: UDP-N-acetyl-glucosamine. Glucosamine may be obtained from exogenous supplements, or it may be endogenously synthesized from glucose through the hexosamine pathway, an alternate pathway to glycolysis. Its product is uridine 5-diphosphate-N-acetyl-glucosamine (UDP-N-Acetyl-GluN), which is a precursor for glycosaminoglycans, proteoglycans, and glycoproteins. (Refer to text.)

This succession of reactions is followed by posttranslational proteic modifications, which have been related to various biological processes, especially those regulating the metabolism of carbohydrates and insulin sensitivity, associated with glucotoxicity and insulin resistance (IR) [42]. Thus, the hexosamine pathway has been proposed to be more than a simple glucosensor, as it may be a potential mediator implicated in the pathogenesis of Type 2 Diabetes Mellitus (T2DM) [35, 43]. The main reactions in the hexosamine pathway are shown in Figure 4.

The rate-limiting step in the regulation of this route involves GFAT, which is the only ammonium-independent enzyme of the amidotransferase subfamily [44]. It is also strongly inhibited by the final product of this metabolic pathway (UDP-N-Acetyl-GluN) through an allosteric mechanism [45]. Therefore, its activity is influenced by UDP-N-Acetyl-GluN intracellular concentrations and intensified by Protein Kinase A (PKA)-dependent phosphorylation [46, 47]. Moreover, its affinity for fructose-6-phosphate is low, so the concentration of this substrate plays an important role in the start-up of this reaction [40].

Ultimately, the plasmatic concentration of glucosamine in healthy subjects is approximately 0.04 mmol/L, rising up to 0.06 mmol/L in those taking common doses of glucosamine supplements [34, 48]. It should be noted that the oral route offers only 20% the plasmatic concentrations which would be obtained through intravenous administration [34, 49, 50]. It has been suggested that the pharmacokinetics and pharmacodynamics of glucosamine in humans closely resemble those of experimental rat models [51].

## 5. Glucosamine: Effective for Osteoarticular Disease?

When evaluating the effectiveness of a drug or therapeutic tool, it is important to consider the target variables susceptible to modification or “end points,” which in the case of clinical assays on patients with knee OA are represented mainly by pain and measurement of articular space [52]. Based on these and other manifestations, several indices or score systems have been created to allow researchers to assess the severity and evolution of the disease when under a given therapy [53, 54], The Western Ontario and McMaster Universities OA (WOMAC) index and the Lequesne functional severity index are some of the most frequently used across various clinical assays [52].

Another aspect worth considering when assessing effectiveness is the type of supplement prescribed; currently, glucosamine hydrochloride and sulfate are the most commercialized in our country and worldwide [55]. However, several studies have reported that when comparing both formulations, glucosamine sulfate exhibits more favorable results, especially in its crystalline form [24, 56, 57]. These differing formulations, as well as differences in pharmaceutical manufacturing, are responsible for distinct pharmacokinetic features which must be taken into consideration, as they could influence comparisons between reports [58].

Regarding dosages, although each presentation shows specific characteristics, therapeutic effects are obtained with doses ranging between 1,250–1,500 mg daily [59, 60]. As their name implies, SYSADOA offer a slow onset of relief—approximately 2 weeks—and their effects may remain active for as long as 2 months after their omission [61]. Notably, the European Medicines Agency has suggested that at least 6 months of treatment are required for the evaluation of SYSADOA effectiveness for articular pain and 2 years are necessary to assess modifications of articular structures [58].

All elements considered a true overarching feature of research in the evaluation of these supplements as their overwhelming heterogeneity with respect to objectives, formulations, combinations with other compounds, and time of use, among many other variables of utmost importance when comparing studies. Indeed, the heterogeneity in outcome measures is particularly noteworthy and unjustified, considering most rheumatologic diseases have been discussed in OMERACT conferences, whose purpose is unification of evaluation criteria for clinical assays in this field [62]. Regarding OA, since OMERACT 3 in 1996, the 3 main aspects to be evaluated in all Phase III studies are pain, physical function, and global patient assessment, as per the simplified OARSI criteria in each of its scenarios and thus allowing for result unification and facilitating comparisons between studies. Only after considering these fundamental aspects can the novel variables in OA progression be considered [52].

This line of research ranges from clinical assays to meta-analyses, encompassing hundreds of patients (Table 1). Parallel studies by Reginster et al. and Pavelká et al. [63, 64] demonstrated the disease-modifying ability of glucosamine sulfate supplements, by ascertaining improvement of symptomatology and prevention of articular space loss in knee OA patients at a 3-year follow-up. Furthermore, results from a subsequent follow-up on these patients at an average of 5 years suggested that treatment with glucosamine sulfate for at least 12 months may prevent the need for knee arthroplasty, revealing the profound extent of the disease-modifying power of this compound [65].

The effects in the short-medium term have been evaluated by studies such as the GUIDE Trial [66], which confirms previous reports regarding the significant improvement glucosamine sulfate yields on symptoms of knee OA, in the range or even superior to what exerted by a first line NSAID or acetaminophen.

Nevertheless, it must be noted that in other reports, benefits do not seem to be present in all analyzed subjects, but only in specific subgroups with distinctive clinical features. This has been exemplified by Clegg et al. [67], who after utilizing glucosamine hydrochloride in their study—a valuable methodological aspect for the comparison of results [58]—could not prove this version of the supplement to reduce pain after 24 weeks in knee OA patients with mild articular pain. These variations in the utilized supplements are indeed very influential. Great-scale research has shown that the use of different commercial brands could factor into results, as suggested by Towheed et al. [31], in their meta-analysis of over 20 randomized controlled trials; only formulations of glucosamine sulfate manufactured by Rotta Laboratories displayed effectiveness in the symptomatic management of patients with OA of the knee, while with other presentations, no statistically significant results were obtained.

In addition to these findings, one of the most controversial reports surrounding the effectiveness of these supplements was issued by Wandel et al. [69], who in their meta-analysis of 10 large-scale randomized controlled trials (3,803 patients) concluded that neither glucosamine nor chondroitin sulfate, neither alone nor combined, could significantly improve pain nor reduction of articular space when compared to placebo, consequently arguing against prescribing these agents in patients with OA. This paper arose numerous criticisms from several specialists and experts in the matter [70, 71], who fundamentally refuted the methodology used in their recollection and analysis of data, as well as their results, sustaining such claims by emphasizing the great variability and heterogeneity of the studies analyzed [58]. Notably, in the report of a post-publication meeting, the BMJ editor withdrew support from the inappropriate conclusions of this meta-nalysis, which were not adequately supported by their data. This illustrates the high degree of controversy attributed to the utilization of these compounds in patients with OA [60].

Although most studies tend to favor the effectiveness of these compounds in subjects with OA at least through minimal or indirect evidence, especially as disease-progression modulators [72], no evidence exists of chondroprotective effects of glucosamine in a preventive context [73]. This fits with the main findings of *in vitro* studies, which suggest a predominantly anticatabolic effect in cell cultures [74]. Several molecular mechanisms are implied, including the inhibition of catabolic enzymes, such as metalloproteases, phospholipase A_2_, and aggrecanase-2 as well as the reversal of the effects of IL-1*β* and cyclooxigenase-2, and inhibition of NF-*κ*B signaling [75–77]. This impact in energetic metabolism and oxidative stress appears to be triggered not only with the consumption of glucosamine alone, but also when accompanied with chondroitin sulfate [78]. These effects have been observed to be more consistent with glucosamine sulfate rather than hydrochloride [79].

Nevertheless, a great proportion of these experimental reports employ glucosamine concentrations much higher than those obtained through the oral ingestion of supplements, hindering the extrapolation of these findings to *in vivo* studies [80]. Regarding studies in animal models, findings are similar to *in vitro* results, with modifications predominantly in synovial inflammation, cartilage degradation, and bone resorption, primarily through the repression of proinflammatory cytokine genes [81]. Ultimately, the heterogeneity in experimental reports resembles its clinical counterpart, with important differences in the types of supplements used, as well as doses and other characteristics which should be unified in future studies. Harmonizing these criteria is a priority in order to accurately and successfully extrapolate molecular mechanisms to human subjects in the clinical scenario.

## 6. Glucosamine: Safety on Glucose Metabolism

Amidst the few adverse effects reported regarding glucosamine supplements [20], the most common are gastrointestinal complaints, including pain, diarrhea, nausea, and pyrosis [82]. On the other hand, although no fulminant events have been described, several cases of allergic reactions have been documented, including angioedema [83], asthmatic crises [84], and photosensitivity [85]. Lastly, much like the controversy surrounding their potential efficacy as therapeutic agents, the consequences of these supplements on carbohydrate metabolism and insulin levels have become one of the most debated topics in rheumatology in recent years [86].

The key point in this matter resides in several findings which associate the hexosamine pathway with the development of IR, with reports as early as the year 1991, when Marshall et al. [35] outlined such a hypothesis after exploring the role of glutamine in their experimental models for IR. As described previously, the final product of this metabolic pathway is UDP-N-Acetyl-GluN (Figure 4), which is precursor for GAG, proteoglycans and glycoproteins. It must be noted that these macromolecules are synthesized in specific cytoplasmic organelles (endoplasmic reticulum and Golgi apparatus) [87], while in the nucleus and cytosol, UDP-N-Acetyl-GluN also serves as a substrate for the enzymatic action of *O*-N-Acetyl-GluN transferase (OGT), which is able to transfer N-Acetyl-GluN to the serine/threonine residues of various proteins in a process known as reversible posttranslational proteic modification [88]. The target proteins of this process include the insulin receptor substrate (IRS) types 1 and 2, as well as GLUT-4 [89, 90]. Numerous research groups suggest, albeit not in a definitive way, that these modifications may represent the molecular basis for IR associated with the hexosamine pathway [87], since it may antagonize the phosphorylation cascade of insulin signaling [42, 90].

Besides these cytosolic mechanisms, *O*-GluNacylation may also target many transcription factors, therefore regulating the expression of proteins in the nucleus [91, 92]. Several transcription factors have been shown to be involved through experimental models as well as genes such as those of glucose-6-phosphatase and phosphoenolpyruvate carboxykinase, key enzymes from the gluconeogenesis pathway [93]. Thus, a great proportion of current research is focused on the role played by OGT in post-translational modifications, as its effects do not seem to be limited to insulin signaling—acting as “metabosensor” mechanism—and it may be part of a wide array of alarm responses or stress reactions in the cardiovascular system [39, 94–96].

Despite these findings in animal models, reports in humans stand divided and although some research suggests metabolic effects for these supplements [50, 104], most clinical trials and meta-analyses suggest this link is not as much clear in humans (Table 2). Moreover, a great part of these studies—including clinical assays [100, 101] and meta-analyses [102, 103]—were carried out on subjects with already impaired glucose metabolism, obscuring the interpretation of their results. Nevertheless, several studies have failed to find associations between GluN administration and insulin resistance as assessed by its gold standard test, the euglycemic insulin clamp (EIC). Such is the case of Monauni et al., with their study on 10 healthy subjects who were assessed through determination of glycemia, application of glucose tolerance test, and the EIC while undergoing glucosamine infusion [97]. Similar results were obtained in 18 healthy individuals with the double forearm technique [98], and no differences in IR nor endothelial dysfunction were evidenced by Muniyappa et al. between 20 lean subjects and 20 obese subjects after short- and long-term (6-week) administration of glucosamine [99].

Certain key points should be noted. Studies discrediting these effects base their conclusions in the magnitude of the required dosage for alterations on carbohydrate metabolism to occur, which must be over 100 times higher than the dose recommended for the management of OA [34]. In addition, long-term studies remain scarce, and current clinical trials suffer from certain methodology errors. Notably, future analyses should categorize and contrast their subjects according to consumption of hypoglycemic drugs and glycemic status, particularly if impaired fasting glucose is present [105]. However, dos Reis et al. [106] have highlighted the safety of crystalline GS regarding not only glucose metabolism but also lipid profile and blood pressure in cohorts from the GUIDE trial and the study by Reginster et al. [63], at follow-ups of 6 months and 3 years, respectively.

Ultimately, determining whether these supplements may influence the metabolism of carbohydrates and insulin secretion in humans should be an imperative objective in the field of diabetology, particularly in the face of recent findings linking glucosamine with endoplasmic reticulum stress [107, 108], a process that under hyperglycemic conditions could trigger a series of deleterious events, including expression of proinflammatory genes, proapoptotic signaling, and lipidic accumulation, which would lead to accelerated atherosclerosis and hepatic steatosis, implying a greater risk for cardiovascular disease [109, 110], besides representing potential pathophysiologic mechanisms for other uncommon adverse events [111].

## 7. Conclusions

Due to the growing impact of OA as a chronic degenerative disease in public health economic systems and the lifestyle of patients, the search for novel therapeutic alternatives must represent a fundamental object for multidisciplinary research groups, including primary attention physicians, rheumatologists, orthopedists, and physiotherapists. Glucosamine supplements, which encompass several types of chemical components, have become a mainstay of OA therapeutic management due to their important structure-preserving and symptom-relieving effects, as well as their cost effectiveness and relatively innocuous adverse effect profiles. Indeed, evidence supporting diabetogenesis as a feasible complication of glucosamine supplement use is scarce, and to date, this association remains only theoretical possibility. Although further research is required to fully understand this relationship, glucosamine supplements have been more than sufficiently proven to display overtly beneficial risk-to-reward profiles, and they should remain fundamental components of OA therapy.

## Figures and Tables

**Table 1:** Studies on the effectiveness of Glucosamine for Osteoarthritis.

Author [reference]	Sample and/or amount of trials	Supplement type, doses	Conclusions
Reginster et al. [63]	Patients treated with GS: 106, placebo: 106	GS (1500 mg OD)	In this randomized, double-blind placebo-controlled trial, treatment with GS prevented loss of articular space and improved symptoms as assessed by WOMAC scoring at a 3-year follow-up.

Pavelká et al. [64]	Patients treated with GS: 101, placebo: 101	GS (1500 mg OD)	This randomized, double-blind placebo-controlled trial also found treatment with GS to prevent loss of articular space and ameliorate pain as assessed by WOMAC scoring and the Lequesne Index at a 3-year follow-up.

Bruyere et al. [65]	Patients treated with GS: 144, placebo: 131	GS (1500 mg OD)	This placebo-controlled prospective study suggests that treatment with GS for at least 12 months and up to 3 years may prevent the need to perform knee arthroplasty in an average follow-up of 5 years after drug discontinuation.

Herrero-Beaumont et al. [66]	Patients treated with GS: 106, acetaminophen: 108, placebo: 104	GS (1500 mg OD), acetaminophen (3 g OD)	In this randomized, double-blind, placebo-controlled study, daily consumption of 1500 mg of GS proved more effective than placebo in the symptomatic management of knee OA. Nonetheless, the effects of acetaminophen were similar.

Clegg et al. [67]	Patients treated with GH: 317, CS: 318, GH + CS: 317, celecoxib: 318, Placebo: 313	GH (1500 mg OD)	In this randomized, double dummy study, neither treatment with glucosamine alone nor combined with CS reduced pain in the average OA patient group during 24 weeks. However, combined therapy may be effective in the group of patients with moderate to severe knee pain.

McAlindon et al. [68]	15 randomized, double-blind, placebo-controlled trials with ≥4 weeks of treatment	GS, GH, or SC versus Placebo	In this meta-analysis, trials indicate these compounds have a moderate to strong effect over OA symptoms, but methodology issues may exaggerate this beneficial effect. Notwithstanding this, they appear to be safe and have a positive impact over symptomatology.

Towheed et al. [31]	25 randomized, controlled trials (4963 patients)	GS (1500 mg OD)	This meta-analysis suggests that GS preparations by Rotta Laboratories may be more effective than placebo in the management of pain and articular functionality as assessed by the Lequesne Index in subjects with symptomatic OA Nevertheless, other formulations did not show any effectiveness (statistical significance) at managing pain nor functionality or rigidity as assessed by the WOMAC scale.

Wandel et al. [69]	10 large scale randomized placebo-controlled trials (3803 patients)	GS, GH, or SC versus placebo	This meta-analysis suggests neither glucosamine nor chondroitin alone, nor their combination, is able to reduce articular pain, nor can they modify articular space in comparison to placebo.

OD: once daily; GS: glucosamine sulfate; CS: chondroitin sulfate; OA: osteoarthritis; GH: glucosamine hydrochloride.

**Table 2:** Studies relating Glucosamine use with the onset of Insulin Resistance.

Author [reference]	Sample and/or amount of trials	Supplement type, doses.	Conclusions
Monauni et al. [97]	10 healthy subjects	Glucosamine (infusion: 1.6 micromol/min^−1^/kg^−1^5 micromol/min^−1^/kg^−1^) **Type not specified**	IVGTT and EIC were performed during either a saline infusion or a low (1.6 micromol) or high (5 micromol) GluN infusion. GluN did not change glucose utilization or intracellular metabolism, nor did it affect readily releasable insulin levels, GSIS, or the time constant of secretion, but it increased both the glucose threshold of GSIS and plasma fasting glucose. These effects were present at high GluN doses.

Pouwels et al. [98]	18 healthy subjects	GS (infusion: 4 micromol/dL)	EIC was performed throughout at least 300 minutes during infusion of GluN (4 micromol/dL); 90–240 min, 0–300 min, or during saline infusion. GluN had no effect on insulin-induced glucose uptake.

Muniyappa et al. [99]	40 lean subjects and 40 obese subjects	GH (500 mg TID) versus Placebo	This study found no differences in EIC between patients receiving GH and placebo after 6 weeks of therapy, within both the lean and obese groups.

Biggee et al. [50]	16 patients with exclusive diagnoses of OA, treated with GS	GS (1500 mg OD)	In this study, carried out in subjects without any metabolic disorders (such as TDM2 and IFG), 3 out of 16 individuals displayed disruption of oral glucose tolerance after treatment with GS. This suggests the necessity for a period of time for this supplements to exert metabolic modifications in this group of patients as well as trials in poorly controlled subjects.

Scroggie et al. [100]	Patients treated with GC: 22, placebo: 12	GH (1500 mg OD) + CS (1200 mg OD) versus Placebo	This randomized, double-blind, placebo-controlled clinical trial carried out in patients with controlled TDM2 determined that the oral adminstration of GH at recommended doses did not alter glycemic control in this group of patients.

Albert et al. [101]	12 patients in a randomized, double-blind, placebo-controlled, cross-over trial	Glucosamine (1500 mg OD) **Type not specified**	This study inferred glucosamine at commonly used doses, may not significatively affect glycemic control, lipid profile, or apoAI levels in diabetic patients after 2 weeks of treatment.

Simon et al. [102]	23 trials with different methodologies: glucosamine IV (infused): 2, oral glucosamine: 21	GS or GH versus placebo	This meta-analysis included trials with both IV and oral formulations, and even long-term reports, concluding that glucosamine consumption at habitual doses may not affect the metabolism of normoglycemic, “prediabetic,” or diabetic subjects and that currently no definitive motives are valid for their restriction in these groups of individuals.

Dostrovsky et al. [103]	11 trials with different methodologies: RCT: 6, prospective studies: 5	GS, GH, or SC versus placebo	This meta-analysis highlighted 3 trials where OA glucosamine was used, followed by modifications in insulin sensitivity and basal glycemia. Additionally, studies that included subjects with IFG or IR showed greater impact over carbohydrate metabolism. Thus, this population should be a target of further research.

IVGTT: intravenous glucose tolerance test; EIC: euglycemic insulin clamp; GluN: glucosamine;. GSIS: glucose-stimulated insulin secretion; TID: three times a day; OD: once daily; GS: glucosamine sulfate; CS: chondroitin sulfate; OA: osteoarthrosis; GH: glucosamine hydrochloride; TDM2: type 2 diabestes mellitus; apoAI: apolipoprotein AI; IFG: impaired fasting glucose; IV: intravenous; RCT: randomized controlled trials; AO: administered orally; IR: insulin resistance.
